# p21 in chronic and acute liver injury

**DOI:** 10.18632/oncoscience.297

**Published:** 2016-03-04

**Authors:** Haksier Ehedego, Christian Trautwein

**Affiliations:** Department of Internal Medicine III, University Hospital RWTH Aachen, Aachen, Germany

**Keywords:** DNA damage, hepatocarcinogenesis, liver injury

p21 historically has been considered a tumor suppressor since first studies showed that p21^−/−^ mice display spontaneous tumor formation after 16 months and additionally these mice are more sensitive to chemically induced carcinogenesis [1,2].

On the contrary, recently a potential function as an oncogene has been described for p21. For instance mice deficient for p53 spontaneously develop multiple tumors and an additional deletion of p21 lead to a significant reduction of thymic lymphomas [3].

This argues that the complete spectrum of p21 function during tumorigenesis is not clearly identified. The role of p21 has been further studied in the NEMO^Δhepa^ mice model. The NF-κB pathway regulator NEMO (also known as IKKγ) has been shown to control chronic inflammation and hepatocarcinogenesis in mice.

The hepatocyte specific deletion of NEMO (NEMO^Δhepa^), is of clinical interest as these animals develop a cascade of events which resemble the spectrum of human chronic liver disease, which leads from chronic hepatitis to liver cirrhosis and growth of hepatocellular carcinomas (HCC). Additionally, a recent study using human HCC tissue found a downregulation of NEMO in tumor tissue, further supporting the translational relevance of the NEMO^Δhepa^ mice model [4].

The deletion of NEMO in hepatocytes triggers increased p21 expression [5,6]. In order to study the relevance of p21 overexpression for disease progression of NEMO^Δhepa^ livers, double knockout (NEMO^Δhepa^/p21^−/−^) mice carrying a hepatocyte specific deletion for NEMO and an additional constitutional deletion for p21 were generated.

Although p21 is a cell cycle inhibitor its deletion had no impact on cell proliferation in 8 week-old NEMO^Δhepa^/p21^−/−^ livers compared to NEMO^Δhepa^ livers. This result was unexpected since p21 binds to CcnE/cdk2 and CcnA/cdk2 complexes thereby preventing progression from G_1_- to S-phase. Very likely the loss of p21 expression is compensated by other cell cycle inhibitors such as p-p27 and p18.

Despite the unchanged cell cycle activity in p21 deficient NEMO^Δhepa^ livers, the cell cycle regulator CcnA2 and CcnE2 were overexpressed. However, recent studies discovered that ectopic overexpression of CcnA or CcnE in mouse embryonic fibroblast (MEFs) lead to an increase in DNA double strand breakage [7]. Therefore the enhanced liver injury caused by exacerbation of DNA damage in p21-deficient NEMO^Δhepa^ mice could be explained by elevated CcnA2 and CcnE2 expression. The DNA double strand breakage was quantified by pH2AX Immunofluorescence staining.

p21 is not only protective against DNA damage in the chronic liver injury model as challenging double mutant NEMO^Δhepa^/p21^−/−^ mice with Lipopolysaccharide (LPS) enhanced DNA damage massively compared to NEMO^Δhepa^ mice. After LPS administration NEMO^Δhepa^ mice suffer from severe liver injury which is reflected in the increased alanine aminotransferase (ALT) and aspartataminotransferase (AST) serum values and apoptotic cells in the liver of these mice. However, in NEMO^Δhepa^/p21^−/−^ mice transaminases and cell death were significantly enhanced. Finally, this enhanced liver injury in the double knockout animals resulted in a higher lethality of this mice after LPS administration.

The observed hypersensitivity against LPS due to the lack of p21 is mediated via the Tumor Necrosis Factor (TNF), since NEMO^Δhepa^/p21^−/−^ mice which carry in addition a deletion for the TNF receptor 1 (NEMO^Δhepa^/p21^−/−^/TNF-R1^−/−^), showed a strong attenuation of the DNA damage and cell death.

The protective role of p21 in carcinogenesis was the first time visible in 26 week old knockout animals. Here, the double knockout mice (NEMO^Δhepa^/p21^−/−^) showed enhanced hepatocyte proliferation as revealed by Ki67 staining. This resulted consequently into a higher liver weight/body weight ratio but more interestingly p21- deficient NEMO^Δhepa^ livers showed more frequently small tumors in comparison to NEMO^Δhepa^ livers.

Finally, a significantly increased number of HCCs were found in 52 week-old NEMO^Δhepa^/p21^−/−^ animals, meaning that the loss of p21 expression caused exacerbation of hepatocarcinogenesis. Analysing the livers of these mice revealed that only the number of nodules was increased, whereas the sizes of the tumors were not significantly enlarged. This suggests that the loss of p21 overexpression in NEMO^Δhepa^ animals has more impact on tumor initiation than on tumor progression.

Beside hepatocarcinogenesis p21 had an additional protective role in cholestasis. Livers of 52 week old NEMO^Δhepa^/p21^−/−^ animals display yellow inclusions and serum values for alkaline phosphatase, direct and total bilirubin confirmed the cholestatic phenotype. These cholestatic serum markers were significantly lower in NEMO^Δhepa^ mice.

Taken together, the enhanced p21 expression in NEMO^Δhepa^ animals has a protective function in this model, as p21 protects against DNA damage, acceleration of hepatocarcinogenesis and cholestasis. Since liver disease progression is reduced in the presence of p21 expression, p21 has been shown to act as a tumor suppressor in the NEMO^Δhepa^ model.
